# Investigating Beliefs in Anti-Vax Conspiracy Theories among Medical Students

**DOI:** 10.3390/vaccines12040359

**Published:** 2024-03-27

**Authors:** Jan Domaradzki, Piotr Jabkowski, Dariusz Walkowiak

**Affiliations:** 1 Department of Social Sciences and Humanities, Poznan University of Medical Sciences, Rokietnicka 7, 60-806 Poznań, Poland; 2 Faculty of Sociology, Adam Mickiewicz University, 60-568 Poznań, Poland; 3 Department of Organization and Management in Health Care, Poznan University of Medical Sciences, 60-356 Poznań, Poland

**Keywords:** attitudes, medical students, anti-vax conspiracy theories, vaccines

## Abstract

While the doctors’ role in immunization is essential, their lack of knowledge or vaccine hesitancy may affect their ability to communicate effectively and educate patients about vaccination, vaccine hesitancy, and vaccine conspiracy theories. This, in turn, may hinder health policy aimed at fighting infectious diseases. Vaccine hesitancy is prevalent not only among the general population but also among healthcare workers; thus, this study is aimed at assessing future doctors’ attitudes towards anti-vax conspiracy theories. A total of 441 medical students at Poznan University of Medical Sciences completed a web-based survey designed to explore their attitudes toward the six most prevalent anti-vaccine conspiracy theories. The survey showed that although over 97% of future doctors support vaccinations as an effective form of fighting infectious diseases, and 80% did not believe in any anti-vax conspiracy theory, a significant fraction of 20% of medical students either believed in at least one such theory or were unsure. It has also shown that male and younger students who had not received a flu vaccination and defined themselves as politically right-wing or conservative and religious were more likely to believe in anti-vax conspiracy theories. Our data suggest that, in order to overcome medical students’ ambivalent attitudes towards anti-vax conspiracy theories, they should receive more education about the importance of vaccination in preventing disease and about effective ways to combat vaccine hesitancy and anti-vax conspiracy theories.

## 1. Introduction

Over the past few decades, we have witnessed a rapid decline in many preventable diseases, including smallpox, measles, polio, diphtheria, tetanus, pertussis, measles, mumps, rubella, rotavirus, and hepatitis-B. While many factors have influenced this process, without a doubt, one of the main reasons for such a decline is vaccinations [1,2,3]. Consequently, vaccination has been defined as one of the greatest public health achievements of the 20th century, both in the United States and the rest of the world [4,5]. However, until recently, the only Nobel Prize for developing a virus vaccine was awarded in 1951 to Max Theiler for discovering an effective vaccine against yellow fever [6]. Then, after more than 70 years, in 2023, another Nobel Prize in Physiology or Medicine was awarded to biochemist Katalin Karikó and immunologist Drew Weissman for the discoveries that enabled the development of effective mRNA vaccines against COVID-19 [7].

Despite all this, suspicions of vaccination are widely believed in many societies [8,9,10,11]. In fact, opposition to vaccination dates back to the origin of the first vaccine, i.e., the cowpox vaccination invented by Edward Jenner, which provoked heated sanitary, religious, scientific, and political objections. For example, while some people believed that because it came from an animal, it was ‘unchristian’ [8]; others suggested that it would make people grow horns [12]. Other objections resulted from general distrust towards the government and the mandatory character of vaccination, as many people believed that introducing unknown substances into one’s body should not be compulsory and violates personal liberty [8,9,10,11]. Consequently, Anti-Vaccination Leagues or Societies were formed in several countries, including the United States, Canada, and the United Kingdom, to abolish compulsory vaccination and fight for personal freedom [10,11].

Unsurprisingly, similar arguments emerged during the discussion on the efficacy and safety of other vaccines, including diphtheria, tetanus, pertussis (DTP), and measles, mumps, and rubella (MMR). However, the anti-vax movement gained momentum in the late 1990s when doctor Andrew Wakefield published a paper claiming that there is a causal link between the MMR vaccine and autism [13,14,15]. Some communities also claim that immunization programs are part of the United States and Israeli governments’ conspiracy aiming at sterilizing Muslim children [16,17]. Similar rumours concerning the polio vaccine occurred in some African and Asian countries, leading to a boycott of polio vaccination campaigns [18,19,20]. Finally, some people raise concerns that the human papilloma virus (HPV) vaccination was designed to cause infertility and prevent overpopulation [21,22].

Unsurprisingly, several vaccine conspiracy theories flourished during the COVID-19 outbreak [23,24,25]. At the same time, most people who oppose the COVID-19 vaccine refer either to the old argument that vaccinations violate human rights to autonomy and personal choice or raise concerns about its safety and efficacy, suggesting that the vaccines were developed too quickly and can lead to many adverse events [26]. There is also a widespread belief that the new generation of mRNA vaccines contains toxic ingredients that can harm or kill people or were even designed for that purpose. Others suggest that the COVID-19 vaccine was intended to modify human DNA and cause infertility to decrease the population of the planet. Another theory claims that either the government or Bill Gates are using COVID-19 vaccines to implant microchips in humans to track and control people [26,27]. Finally, some minority groups, including the Nation of Islam, argued that vaccination is a part of systematic ‘medical racism’ aiming at sterilization of the Muslim community [17,28].

Although it is often suggested that people who support anti-vaccine conspiracy theories tend to be less educated and wealthy, religious, right-wing, and members of minority groups, their supporters come from across the social and political spectrums [29,30]. Moreover, while COVID-19 vaccine hesitancy especially is prevalent among the general public, research suggests that it is also high among healthcare workers and medical and healthcare students [31,32,33,34,35,36,37,38,39,40,41,42]. The issue is of crucial importance because while healthcare workers’ attitudes may enhance the public’s willingness to vaccinate, if doctors exhibit insufficient knowledge or express vaccine hesitancy and do not educate their patients about vaccine recommendation and evidence-based information on its benefits for individual and public health, or even spread suspicions towards vaccines, they may hinder health policy aiming at fighting infectious diseases which are currently on the rise, including future epidemics, especially since people who believe in anti-vaccine conspiracy theories turn to the Internet and are less likely to rely on family physicians and follow recommended prevention strategies, such as practicing hand hygiene, wearing masks, keeping social distance, and getting tested and vaccinated [43,44,45]. This is particularly important because Poland is among the countries with the lowest acceptance of vaccinations against COVID-19 [46]. Similarly, the vaccination rate against influenza is systematically decreasing in Poland and is one of the lowest in Europe [47], and the HPV vaccination coverage rate in the country does not exceed 15–20% [48].

Thus, since medical universities are forges of future healthcare workers who should be able to communicate with their patients about vaccination, vaccine hesitancy, and vaccine conspiracy theories [49,50,51], this study, therefore, seeks to explore future doctors’ attitudes towards anti-vaccine conspiracy theories. Although only recently several studies on Polish medical students’ COVID-19 vaccine hesitancy have been conducted [52,53,54], they often focus on students’ knowledge and willingness to get vaccinated. However, while several factors that may influence such decision were identified, including poor knowledge, fear of vaccine side effects, or having contact with anti-vaccination propaganda, still, little is known about future doctors’ support for anti-vaccine conspiracy theories. Meanwhile, since in the future medical students will play a crucial role in prevention of infectious diseases and promotion of vaccinations, their vaccine hesitancy may affect their ability to mitigate patients’ concerns and the spread of vaccine negationism. Thus, we argue that while future doctors support vaccinations as an effective form of infectious diseases and their acceptance for vaccine negationism is low, still the level of vaccine hesitance is moderately high.

## 2. Methods

### 2.1. Study Design

This study presents the data from a self-administered, anonymized web survey of medical students enrolled at the Poznan University of Medical Sciences (PUMS), which is one of the best and largest medical universities in Poland, ranked sevenths among Polish medical universities in the county, and 79th of all 2730 universities from the Central and Eastern Europe region [55]. While PUMS owns five clinical hospitals, it is also known for having one of the best medical courses and research centers in Poland, and is important for providing quality health services to the local community. As a public medical university, PUMS is fully recognized by the Polish Ministry of Health, and Ministry of Science and Higher Education. Additionally, as it is a part of the European Higher Education Area it conforms to the guidelines the Bologna Process, ad its diplomas are recognized and accepted in the European Union and worldwide.

At the same time, while there are some tools for the assessment of people’s conspiracy mentality [56] or vaccine conspiracy beliefs [57], most frequently they focus on the assessment of people’s tendency to engage in conspiracist ideation or their knowledge on vaccinations and health care provider recommendations and one’s willingness to vaccinate, but rarely do they ask questions about specific anti-vax conspiracy theories. Thus, because there is no particular tool for assessing people’s support for a range of anti-vax conspiracy theories, drawing from a literature review [8,9,10,11,12,13,14,15,16,17,18,19,20,21,22,23,24,25,26,27], we have constructed an original questionnaire. We believe that it will help us to gain a more in-depth knowledge about types of anti-vaccine conspiracy theories that should be particularly targeted during medical education and training.

### 2.2. Research Tool

The questionnaire used for this study was designed according to the guidelines of the European Statistical System [58]. It was consulted on by two external experts in public health and medical sociology. Next, it was pre-tested in a pilot study via an online communication platform used by students at PUMS for educational purposes (Microsoft Teams) with a group of 20 students, which led to reformulating two questions.

The questionnaire used closed-ended, single-choice questions to explore medical students’ beliefs in vaccine conspiracy theories, and it comprised two domains. The first addressed medical students’ demographic characteristics. Apart from standard questions concerning sex, year of study, faculty, domicile, and declared religiosity, it also included questions regarding respondents’ medical history (i.e., previous hospitalizations, experiencing chronic disease, being a blood or marrow donor, being vaccinated against COVID-19, receiving a flu vaccination), declared trust in science and medicine and political orientation. The second domain included questions regarding the seven statements on vaccinations: one referring to the effectiveness of vaccination in fighting infectious diseases, and six statements regarding most prevalent anti-vax conspiracy theories.

The final version of the questionnaire comprised seventeen questions.

### 2.3. Participants and Setting

Students were recruited through an online platform used at PUMS. While convenience sampling was used in this study, the following inclusion criteria were used: participants had to be enrolled at PUMS, study medicine, be willing to participate in the study, provide written informed consent before completing the survey, and be able to use the Internet to participate.

### 2.4. Data Collection

The survey was conducted among medical students at PUMS between December 2023 and January November 2024. Before completing the study, all students were informed by the research coordinator (JD) about the study aim and the survey’s voluntary, confidential, anonymous, and non-compensatory nature. They were also informed about the possibility of quitting the questionnaire at any time without any consequences. Once written consent was obtained from all participants who agreed to complete the survey, all students received the link to the questionnaire posted online. Students completed the survey using electronic devices. It took between 5 and 7 min to complete the survey.

### 2.5. Ethical Issues

This study was designed and conducted in line with the principles of the Declaration of Helsinki (revised in 2000) [59]. It was also approved by the PUMPS Bioethics Committee (KB–1075/24, granted on 16 January 2024). All students who agreed to participate in the study signed an online informed written consent form to participate before completing the survey.

### 2.6. Data Analysis

**Figure 1:**
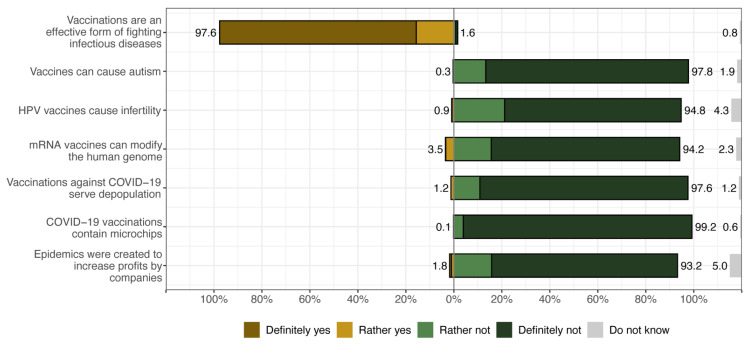
Medical students’ support for anti-vax conspiracy theories.

**Figure 2:**
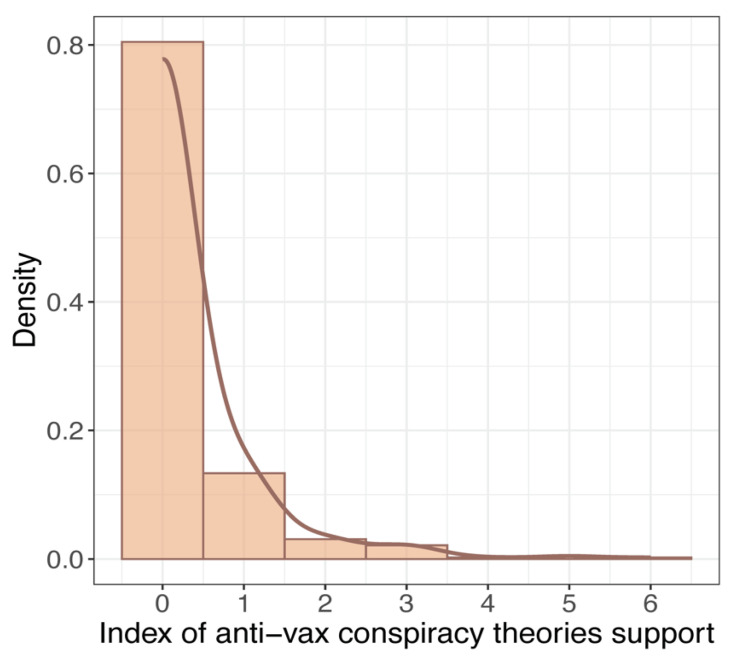
Histogram and density plot for the index of support for anti-vax conspiracy theories.

Our core analysis incorporates a series of regression models to assess whether receiving the flu vaccination, respondents’ medical history, the role of religion in life, and political orientation correlated with the propensity to believe in anti-vax conspiracy theories. As we found that overall, beliefs regarding anti-vax conspiracy theories were extremely skewed toward the rejection of conspiracy theories (see the Section 3 Results below; Figure 1 and Figure 2), we decided to estimate bivariate logistic regressions (instead of ordinal logistic regression) by contrasting those who do not believe in any of anti-vax conspiracy theories with those who believe in at least one. Thus, the dependent variable ${antiVax}_{i}$ takes two values, such that $E\left({antiVax}_{i}=1\right)=\pi_{1i}$ is a probability that student i will believe in at least one anti-vax conspiracy theory, and $E\left({antiVax}_{i}=0\right)={1-\pi }_{1i}$ is the probability of the opposite event. Additionally, we transformed the abovementioned probabilities by the logit link function, where the logit coefficient $\eta_{i}=log\left(\pi_{1i}/\left({1-\pi }_{1i}\right)\right)$ is the log of the odds of the event ${antiVax}_{i}=1$, as opposed to ${antiVax}_{i}=0$.

We built different regression models, with covariates and controls added stepwise. Thus, we ran series of reduced and adjusted models; in the latter, we controlled for the effect of selected sociodemographic characteristics of respondents on the covariates as detailed in Table 2 in the Section 3 results. We opted not to include all covariates and control variables in a single model, considering an events-per-variable criterion to determine the number of variables that could be included in the regression to achieve a minimally acceptable level of statistical power [60] and to avoid the risk of collinearity of some covariates [61]. Our assumed logistic regression model was as follows:

$$\eta_{i}=\beta_{0}+\beta_{j}\Lambda_{i}^{j}+\beta_{k}\Gamma_{i}^{k}$$

where $\beta_{0}$ is an intercept, $\beta_{j}$ is a vector of regression coefficient on all covariates expressed as $\Lambda_{i}^{j}$ (i.e., receiving a flu vaccination, previous hospitalizations, experiencing chronic disease, being a blood or marrow donor, being vaccinated against COVID-19, receiving a flu vaccination, declared trust in science and medicine, the role of religion in life, and political–normative orientation, respectively), and $\beta_{k}$ denotes a vector of regression coefficients on control variables expressed as $\Gamma_{i}^{k}$ (including sex, year of study, and domicile). Note that in reduced models, the matrix of control variables is empty.

All analyses were performed in the R Project for Statistical Computing [62]. We implemented the following packages for data analyses and visualization: *tidyverse* [63], *haven* [64], *ggplot2* [65], *sjPlot* [66], and *flextable* [67].

## 3. Results

Table 1 shows the characteristics of the study participants. Out of all 2321 medical students comprising the population, 441 participated by completing the survey, providing a response rate of 17.7%. The sample consisted of 270 women (65.7%) and 139 men (33.8%), all of Polish origin. While study participants represented various years of study, 57.2% were enrolled in their first or second year of study, whereas 42.8% studied in the third, fourth, or fifth year. Note that as we had information on the size of the population, i.e., the number of students studying in a consecutive year by field of study, we used this information to calculate weights that adjust for differences between the sample and the population in terms of both characteristics.

Most study participants lived in big towns with more than 500,000 inhabitants (40.9%) or in towns with fewer than 100,000 inhabitants (45%). While 28% of students declared that religion influences their life decisions and choices, 72% claimed that religion is irrelevant. A total of 21.7% of respondents reported being hospitalized within the past five years, and 33.3% suffered from some chronic disease. Slightly above 23% reported blood donation experience, and 27.7% were declared bone marrow donors. While nearly all students declared being vaccinated against COVID-19 (97.8%), fewer than half claimed receiving the flu vaccination (41.8%).

Most respondents described both their political attitudes and worldview beliefs as leftist (51.3% and 68.9%, respectively) or centre (35.8% and 19.5%, respectively), while approximately 12% defined themselves as rightist/conservative (12.9% and 11.7%, respectively).

Our analysis begins with descriptive statistics examining the distribution of students’ beliefs in vaccine conspiracy theories. Note that study participants were presented with seven statements and asked whether they agreed or disagreed with them. The response options were in an agree–disagree format, namely ‘definitely not’, ‘rather not’, ‘rather yes’, and ‘definitely yes’, with additional ‘don’t know’ responses treated as the midpoint of a response scale. The first statement was general and measured whether students believed that (1) vaccinations are an effective form of fighting infectious diseases, while the remaining six statements described various specific vaccine conspiracy theories, i.e., that (2) vaccines can cause autism, (3) HPV vaccines cause infertility, (4) mRNA vaccines can alter the human genome, (5) vaccinations against COVID-19 are for depopulation, (6) COVID-19 vaccinations contain microchips, and (7) the Zika, Ebola, MERS, and SARS-CoV-2 epidemics were created to increase the profits of biotechnology companies. Figure 1 shows the percentage of respondents who agree and disagree with the seven statements related to anti-vax conspiracy theories.

When analysing the survey responses regarding various vaccine-related beliefs presented in Figure 1, it is clear that there is a widespread consensus among medical students regarding the effectiveness of vaccines in combating infectious diseases, with over 97% of respondents expressing that they ‘definitely’ or ‘somewhat’ believe in their effectiveness. Conversely, the same proportion of participants do not attribute a link between vaccines and autism, expressing a ‘definitely’ or ‘somewhat’ disagreement, and a similarly high proportion of medical students (over 90% in each case) do not attribute HPV and mRNA vaccines with altering the human genome and do not believe that COVID-19 vaccines are for depopulation, contain microchips and that epidemics such as Zika, Ebola, MERS, and SARS-CoV-2 were created to increase profits for biotechnology companies. However, even though the fraction of medical students who reported believing in anti-vax conspiracy theories is small, a non-negligible fraction of them ‘don’t know’ whether or not to accept conspiracy theories related to vaccinations.

Based on students’ ratings of whether they agreed or disagreed with the above statements, we constructed an index of support for anti-vax conspiracy theories. First, we dichotomized the ratings of the statements by contrasting those who answered ‘definitely yes’, ‘rather yes’, or ‘don’t know’ (score 1) with the rest of the respondents (score 0), except for the first statement, where we contrasted those who answered ‘definitely not’, ‘rather not’, or ‘don’t know’ (score 1) with the rest of the respondents (score 0). Thus, for each item, a score of 1 means that the respondent believes in a specific anti-vaccine conspiracy theory or does not believe in the efficacy of vaccination in controlling infectious diseases in general, and a score of 0 means the remaining counterpart. Second, for each respondent, we calculated the sum of the scores for each of the seven items; thus, the index ranges from 0 to 7 and counts how many anti-vax conspiracy theories students believe. Figure 2 shows a histogram and density plot of the distribution of the index scores. Note that 80% of all study participants do not accept any anti-vax conspiracy theory, which means that 20% of medical students believe in at least one.

**Figure 3:**
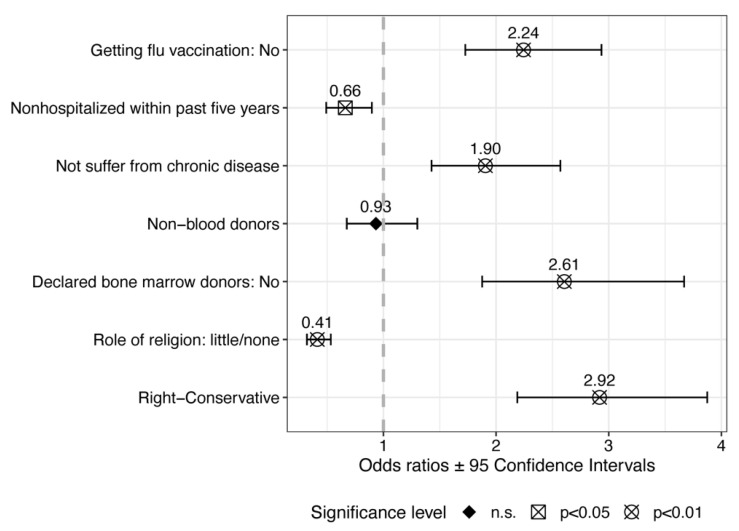
Summary of regression results for reduced models. n.s. Response option not selected.

Figure 3 summarizes the results of five different reduced logistic models, with belief in anti-vax conspiracy theories as the dependent variable. All the covariates were added stepwise. Model 1 includes only flu vaccination, model 2 includes students’ medical history—hospitalization and suffering from chronic diseases, model 3 includes blood donation and bone marrow donation as covariates, model 4 includes the role of religion, and model 5 includes the political and normative orientation as the only covariate. In addition, the regression coefficients have been transformed using the exponential function; thus, a value greater than 1 (vertical dashed line) indicates that the predictor increases the likelihood of believing in anti-vax conspiracy theories. The shape of the points indicates whether the regression coefficient is statistically significant at *p* < 0.05 or *p* < 0.01, respectively.

The results of the reduced regressions demonstrate that medical students who reported not having had the flu vaccine were 2.2 times more likely to believe in anti-vax conspiracy theories than those who had had the flu vaccine. Similarly, those who declared they do not suffer from chronic disease and those who said they were not bone marrow donors as well as those who declared as politically right-wing and conservative were also significantly more likely to believe in anti-vax conspiracy theories. Conversely, students who reported they had not been hospitalized within the past five years and who declared that religion plays little or no role in their lives had significantly lower odds ratios of believing in anti-vax conspiracy theories compared to their religious counterparts.

Table 2 presents the results of the adjusted logistic regression models, i.e., with sociodemographic characteristics added to control for the relationships observed in reduced models (again, the regression coefficients were transformed to represent odds ratios for each explanatory variable). The table also includes information on whether the regression coefficients for the regression terms were statistically significant by reporting detailed *p*-values, and we calculated standard errors for the following coefficients. In addition, we performed the likelihood ratio test (LRT) for each model to assess whether the model’s reduction in deviation was statistically significant compared to the null deviation. We found the reduction to be significant for each model (for details on the model fit statistics, see the *p*-values obtained in the LRT test, along with the Akaike Information Criterion score and log-likelihood shown at the bottom of Table 2).

**Table 2:** Summary of regression results for adjusted models.

Predictors	Model 1		Model 2		Model 3		Model 4		Model 5	
	OR (SE)	*p*	OR (SE)	*p*	OR (SE)	*p*	OR (SE)	*p*	OR (SE)	*p*
Intercept	0.30 (0.05)	<0.001	0.54 (0.12)	0.004	0.30 (0.07)	<0.001	0.88 (0.15)	0.448	0.41 (0.06)	<0.001
Gender: Male [vs. Female]	1.25 (0.17)	0.110	1.12 (0.16)	0.418	1.10 (0.17)	0.189	1.17 (0.16)	0.267	1.04 (0.15)	0.798
Year of study 3–5 [vs. 1–2]	0.48 (0.10)	0.001	0.44 (0.06)	<0.001	0.52 (0.07)	<0.001	0.43 (0.06)	<0.001	0.40 (0.05)	<0.001
Domicile: 10–100 k [vs. Up to 10 k] inhabitants	0.56 (0.10)	0.001	0.52 (0.09)	<0.001	0.53 (0.09)	<0.001	0.48 (0.09)	<0.001	0.50 (0.09)	<0.001
Domicile: 100–500 k [vs. Up to 10k] inhabitants	0.67 (0.14)	0.058	0.62 (0.13)	0.026	0.65 (0.14)	0.039	0.73 (0.15)	0.131	0.70 (0.15)	0.085
Domicile: <500 k [vs. Up to 10 k] inhabitants	0.38 (0.06)	<0.001	0.36 (0.06)	<0.001	0.38 (0.06)	<0.001	0.39 (0.06)	<0.001	0.39 (0.07)	<0.001
Received flu vaccination: No [vs. Yes]	1.93 (0.27)	<0.001								
Hospitalized within past five years: No [vs. Yes]			0.59 (0.09)	0.001						
Suffering from chronic disease: No [vs. Yes]			1.61 (0.25)	0.002						
Blood donor: No [vs. Yes]					0.84 (0.14)	0.322				
Declared bone marrow donor: No [vs. Yes]					1.99 (0.36)	<0.001				
Role of religion: Little/none [vs. Important]							0.41 (0.06)	<0.001		
Right–conservative [vs. Left–liberal]									3.06 (0.47)	<0.001
Observations	401		401		401		401		401	
Likelihood Ratio Test (*p*-values)	<0.001		<0.001		<0.001		<0.001		<0.001	
AIC	1574.322		1582.870		1583.939		1555.988		1548.729	
log-Likelihood	−780.161		−783.435		−783.969		−770.994		−767.364	

Regarding Model 1, in which three sociodemographic characteristics of the respondents were included to control the effect of receiving a flu vaccination, we observed a significant relationship observed for this covariate. Moreover, when analysing regression coefficients for the socio-demographic variables, we noted that, on average, respondents in their 3rd to 5th year of study were significantly less likely to believe in anti-vax conspiracy theories (the odds ratio is slightly below 0.5; i.e., the propensity is halved compared to their counterparts in their 1st and 2nd year of study) (Table S1 shows the percentage of students agreeing with each of the anti-vaccine theories by year of study).

Similarly, the tendency to believe in anti-vax conspiracy theories was also halved for respondents living in cities with between 10,001 and 100,000 inhabitants and even lower for those living in large cities (i.e., over 500,000 inhabitants) compared to the reference category, i.e., those living in a small area of residence (up to 10,000 inhabitants). At the same time, for those living in cities with between 100,001 and 500,000 inhabitants, the results were mixed and depended on the model applied. In addition, the regression results show that male and female respondents did not differ significantly, although male respondents were slightly more likely to believe in anti-vax conspiracy theories. Note that the associations for the control variables remain significant in all subsequent models.

In Model 2, respondents’ medical history (i.e., whether the student has been hospitalized in the past five years and has a chronic illness) was included in the regression along with the sociodemographic variables. We found evidence of a significant relationship between these two covariates and the dependent variable, as indicated by *p*-values below the critical level of 0.01. In turn, in Model 3, no evidence was found of a significant effect (at the *p*-level equal to 0.05) of blood or bone marrow donors, even though those who reported not being registered as bone marrow donors were found to support anti-vax conspiracy theories more than twice as often as those who reported being in a bone marrow donor registry.

Finally, Model 4 and Model 5 include the role of religion in life and political orientation as covariates, respectively. We found significant regression coefficients in both cases and the expected direction. Those who reported that religion plays little or no role in their lives have significantly lower odds ratios of believing in anti-vax conspiracy theories compared to medical students who said that religion plays an important role. On the other hand, students who identify themselves as politically right-wing and conservative are almost two and a half times more likely to believe in anti-vax conspiracy theories than students who identify themselves as politically left-wing and liberal in terms of norms and values.

## 4. Discussion

During the pandemic, numerous studies have delved into the reasons for the low interest of medical school students in vaccinations. Most of them did not explore the issue from the angle of conspiracy theories. However, results from various countries revealed that not all students and medical professionals were vaccinated or expressed intentions to be vaccinated [68,69]. In recent years, there has been growing concern about the impact of conspiracy theories, heightened further by the COVID-19 pandemic, especially during the first wave of novel coronavirus when, due to lockdowns, many people turned to the Internet and social media, which become their major source of information. However, it was shown that such platforms as Facebook, Twitter, and YouTube contributed significantly to the dissemination of COVID-19-related conspiracy theories, becoming a source of the ‘infodemic’ and mass suspicions about vaccination [70,71,72,73]. The outbreak has witnessed the widespread dissemination of numerous conspiracy theories, posing a potential threat to adherence to recommended preventive measures. Until November 30, 2020, Islam et al. identified 637 items of COVID-19 vaccine-related social media information, of which 91% were rumors and 9% were conspiracy theories [26]. These items comprised news articles, social media narratives, online reports, and/or blogs that garnered approximately 103.3 million likes, shares, reactions with emojis, or retweets on social media.

The emergence of various conspiracy theories during the pandemic raises concerns, particularly due to documented evidence indicating their negative impact on vaccination intentions [43,73,74,75], even among healthcare workers [31,32,33,36,37]. Mistrust in medical information was also a factor influencing this state of affairs, as indicated [76]. For aspiring medical professionals, the situation is further complicated, as these conspiracy theories not only affect their personal vaccination decisions but also have the potential to influence how they approach persuading patients to get vaccinated. Finally, it is worth noting that conspiracy theories concern not only vaccinations, prompting questions about how a future doctor’s professional activities and beliefs can be influenced. This is even more important because in our study, almost 20% of medical students believe in at least one anti-vax conspiracy theory or were unsure.

While the majority of people in any country do not believe in misinformation about COVID-19, certain misinformation claims are consistently regarded as reliable by a significant portion of the public, posing a potential risk to public health. Importantly, a clear link has been demonstrated and replicated internationally, showing that susceptibility to misinformation is associated with vaccine hesitancy and a decreased likelihood of adhering to public health guidance [77].

Research on medical students’ knowledge about vaccination indicates a generally moderate level of knowledge and positive attitudes, with identified gaps, especially in revaccination and flu vaccination [38,39,40,41]. These gaps suggest a need for additional education and training in these specific areas. The studies also underscore the influence of the year of study and experience in delivering immunizations on knowledge levels, hinting at the potential impact of targeted interventions in medical education [42].

Our study found that the vast majority of medical students believe vaccinations are an effective means to combat infectious diseases. However, a small percentage (1.9%) in our surveyed group disagrees, and an even smaller percentage (0.6%) has no opinion on the matter. While these numbers represent only a handful of students in our sample, their very existence remains intriguing. It is noteworthy that such views also exist among medical specialists; Sule et al. identified 52 physicians in 28 different specialties involved in propagating COVID-19 misinformation [78].

Consistent research findings indicate the detrimental impact of anti-vaccine conspiracy theories on vaccination intentions [43]. However, this influence can differ across groups, as demonstrated by higher vaccination intentions among Polish medical students compared to their non-medical counterparts [52]. Regardless of their major, Polish students exhibit considerable knowledge gaps in vaccination and require additional education on this subject. This knowledge deficit extends to the COVID-19 pandemic, where medical students display a greater willingness to receive the SARS-CoV-2 vaccine compared to other students [68]. However, it is worth noting that Polish medical students exhibit a more positive attitude toward vaccination than nursing students [53]. Jastrzębska et al.’s research results suggest that vaccination knowledge may not be optimal among Polish medical students [54]. Our study findings further suggest a potential connection between belief in conspiracy theories and attitudes contrary to influenza vaccination. There is no such connection with COVID-19 vaccinations because students were obliged to be vaccinated during the pandemic, which resulted in an increase in the vaccination rate, probably sometimes against the wishes of the vaccinated themselves. Flu vaccinations are voluntary, and we are able to determine the relationship between vaccination and belief in conspiracy theories. The influenza vaccination rate in the study group was not high, but belief in one of the conspiracy theories seems to have figured heavily in the decision to get vaccinated against influenza or not.

During the COVID-19 pandemic in Poland, vaccine hesitancy was particularly pronounced among certain groups. Research and reports have indicated that vaccine hesitancy was prevalent in Poland, with some experts attributing it to decades of Communist rule that eroded public trust in state institutions. The country had one of the lowest rates of double-jabbed citizens in the European Union, and vaccine hesitancy was particularly pronounced in Central and Eastern Europe [79].

Additionally, a study evaluating the approach towards vaccination against COVID-19 among the Polish population found that the rapid development of COVID-19 vaccines presented a significant challenge to their general acceptance, including in Poland. The study aimed to target groups of Polish citizens with the highest risk of vaccine hesitancy, indicating that certain segments of the population were more hesitant towards COVID-19 vaccination. Therefore, it can be inferred that vaccine hesitancy was prevalent among certain segments of the Polish population during the COVID-19 pandemic [29].

While there is a common notion that individuals endorsing anti-vaccine conspiracy theories are typically less educated, religious, right-wing, or associated with minority groups, it is noteworthy that proponents of such theories come from various social and political backgrounds. Polish research, akin to studies from other countries, has highlighted the link between vaccine hesitancy and the influence of right-wing political ideologies opposing vaccination in diverse forms [29,30,79,80,81,82,83], as well as religious beliefs [30,84]. The effect size of these findings is substantial and warrants significant consideration. Our study demonstrated that the surveyed group of medical students mirrors societal profiles in this regard; we also observed associations between belief in conspiracy theories, a heightened role of religion in individuals’ lives, and more frequent conservative political affiliations. Furthermore, attention should be drawn to the correlation between belief in conspiracy theories and being a bone marrow donor, as well as being free of chronic diseases; these results also exhibit a considerable effect size.

An earlier study [85] demonstrated that students who declared having been exposed to information posted on social media suggesting not to receive the flu vaccination had a higher level of vaccine hesitancy. While Jamison et al. who analyzed 2000 most active Twitter accounts discussing vaccines, identified both vaccine opponents and proponents, they also found that 17% of them were bots [86]. Simultaneously, 35.4% those who opposed vaccines shared unverifiable or even fake information, including anti-vax conspiracy theories, coronavirus myths, scams and rumors.

Previous studies have demonstrated that although vaccine sceptics and negationists constitute a minority among the public which in general favors benefits of vaccination, they exert a significant online presence, particularly on Twitter. In was also shown that in particular, messages on vaccines contribute to the dissemination of misinformation and disinformation on public health issues on social media [87,88]. Furthermore, vaccine skepticism and negationism is mainly spread by such threat actors as bots and state-sponsored trolls who were promoting vaccination misinformation in cyberspace even well before the COVID-19 outbreak [86].

Twitter bots and trolls, particularly those associated with Russia, play a substantial role in shaping online conversations about vaccination over the years. These entities exhibit a higher frequency of posting vaccination-related content compared to regular users. Interestingly, the content they share reflects an equal emphasis on both pro- and anti-vaccination perspectives, aligning with a strategy to sow discord on contentious issues—a tactic commonly observed in Russian troll accounts [87]. One notable example is the Russian Internet Research Agency, founded in 2014 and aimed at influencing on the Internet by disseminating online propaganda, including fake news about the safety of vaccines [89]. For example, it was demonstrated that while such misinformation tweets rarely contain references to reliable sources, they use anti-vaccination language, refer to various symptoms and personal dangers which are already stressed in headlines, and frame vaccines as a threat to personal liberty [90,91].

Today, social media is an important source of information, and there is an even greater need for an appropriate information policy capable of dealing with disinformation. This applies not only to societies as such but also to individual groups, including medical professionals. How else can we explain the over 6% of students who have no opinion about whether HPV vaccines cause infertility, or the 2.2% who have no opinion about vaccines causing autism, and the few people who think this is the case?

A positive conclusion from our study is that the difference between students of younger and older years of study was captured, and older students are less susceptible to the influence of conspiracy theories. This is in line with previous findings [45,85,92,93], as well as with Baessler et al., who additionally drew attention to the low perceived status of teaching on the topic of vaccinations, especially in the earlier semesters [94]. However, in the study by Lo Moro et al., as many as 8% of surveyed medical students did not feel adequately informed about vaccination [95].

It seems that in light of our results, which show a large group of medical students who believe in, or even more often do not strongly reject, conspiracy theories, changes in the content of teaching in this area seem necessary to ensure the proper quality of education. A similar conclusion was drawn by Gautier et al., examining vaccine hesitancy among French healthcare students, who found it to be high [35]. Similarly to our study, in the case of belief in conspiracy theories, in their case of vaccine hesitancy, a dependence on the year of study was noticed, which should be considered at least a partial success of university teaching. Similar results were obtained in Saudi Arabia by Habib et al., where the difference in vaccine hesitancy for pre-clinical and clinical students was also huge [92], and by Jamil et al. in Pakistan. It seems that reliable medical knowledge is, to a large extent, an effective tool in the fight against vaccine hesitancy and conspiracy theories [96].

The crucial role of scientists in disseminating accurate and reliable information was emphasized, along with the potential significance of promoting numeracy and critical thinking skills to mitigate susceptibility to misinformation [77]. Misinformation surrounding COVID-19 and vaccines, including rumors and conspiracy theories, should not be viewed solely as beliefs in false theories. Rather, they can be interpreted as manifestations of widespread fears and anxieties [23]. These narratives often arise during periods of heightened social uncertainty, and this is exactly the world we will live in now. Medical students listen to them because they appear in public discourse, and everyone has contact with them, whether they want it or not. The aim of a sensibly conducted health policy should not be to prevent their occurrence, but to deal with them at the scientific level, explaining untruths and simplifications, refuting false myths, as well as providing effective medical education based on scientific facts.

This study is not without limitations. Firstly, although 441 medical students responded and completed the survey, the sample size was still relatively small. Secondly, this research was conducted among medical students at only one Polish university and has a local dimension. For both these reasons, our results represent solely the opinions of those students who completed the questionnaire and cannot be generalized to the entire population of medical students either in Poznan or in Poland. Thirdly, since this study used a quantitative method only, it would be desirable to conduct a more in-depth study using qualitative methods that would help to understand medical students’ opinions on anti-vaccination conspiracy theories. Fourthly, although the questionnaire used for this study was consulted on with two external experts in public health and medical sociology and was pre-tested in a pilot, it was not validated. Fifthly, because this study was designed as an online survey and was conducted via a communication platform used at the PUMS, there is a risk that some students, especially those in their clinical years, failed to receive an invitation.

Despite these limitations, however, the unquestionable strength of this study is that, since there is a scarcity of studies describing the attitudes of medical students towards anti-vax conspiracy theories, it may help to stimulate the discussion on the role of future doctors in enhancing the public’s willingness to vaccinate and overcome vaccine hesitancy. Additionally, by identifying the factors associated with medical students’ beliefs in anti-vax conspiracy theories, this study may also help identify steps that should be undertaken in medical education in order to strengthen their ability to communicate with patients. Finally, carrying out this type of research among students and then presenting them the results obtained could have an educational value and in itself lead to a reduction in the frequency of succumbing to conspiracy theories.

## 5. Conclusions

Although this study has shown that the vast majority of future doctors support vaccinations as an effective form of fighting infectious diseases, and 80% did not believe in any anti-vax conspiracy theory, a significant fraction at 20% of medical students either believed in at least one such theory or were unsure. This is important because while doctors are the most trusted source of information about vaccines, and their role in immunization is essential, medical students’ vaccine hesitancy may affect their ability to communicate effectively with patients about vaccination, vaccine hesitancy, and vaccine conspiracy theories. This may, in turn, hinder their role as medical educators. We therefore suggest that, although during immunology classes at PUMS, medical students are taught about vaccine development strategies and different types of vaccines and immunization, and during their health promotion course they are trained in epidemiology of infectious diseases, the importance of vaccination in disease prevention and their benefits for personal and public health, recommended vaccinations and immunization schedule, and vaccine hesitancy, they should be also trained about the importance of social media in shaping social attitudes towards vaccination and dissemination of anti-vaccine conspiracy theories. This is particularly important since research has shown that using social media influence individuals’ ability to identify misinformation, their support for anti-vaccine conspiracy theories and willingness to get vaccinated [97,98,99]. Additionally, since there are numerous factors that affect people’s vaccine hesitancy, including, medical, cognitive, psychological, social, cultural, and religious reasons, students should be also familiarized with differences in patients’ perceptions of vaccination, including possible differences related to social conditioning. Moreover, because during their clinical practice future doctors face variety of vaccine mindsets, they should be trained in patient-centered discussion about vaccines. Thus, they need to develop skills that will help them to personalize their approach for discussing vaccine hesitancy and anti-vaccine beliefs. One possible solution to develop and enhance such skills is to organize discussions with patients through a web-based educational curriculum where they could discuss on important concepts in the fields of public health as well in a humanist and ethical approach to health.

## Figures and Tables

**Table 1:** Study participants.

Characteristics	Total (n = 411)
*Sex:*	
Female	270 (65.7%)
Male	139 (33.8%)
Did not answer	2 (0.5%)
*Year of study*	
1st–2nd year	235 (57.2%)
3rd–5th year	176 (42.8%)
*Domicile:*	
Up to 10,000 inhabitants	90 (21.9%)
10,000–100,000 inhabitants	95 (23.1%)
100,000–500,000 inhabitants	58 (14.1%)
More than 500,000 inhabitants	168 (40.9%)
*Role of religion in life*	
Important	115 (28.0%)
Few	122 (29.7%)
None	174 (42.3%)
*Being hospitalized within the past five years*	
Yes	89 (21.7%)
No	322 (78.3%)
*Suffering from any kind of chronic disease*	
Yes	137 (33.3%)
No	274 (66.7%)
*Blood donation*	
Yes	96 (23.4%)
No	315 (76.6%)
*Declaration of marrow donation*	
Yes	114 (27.7%)
No	297 (72.3%)
*Vaccination against COVID-19*	
Yes	402 (97.8%)
No	9 (2.2%)
*Getting the flu vaccination*	
Yes	172 (41.8%)
No	239 (58.2%)
*Left–right orientation (political attitudes)*	
Left	211 (51.3%)
Centre	147 (35.8%)
Right	53 (12.9%)
*Liberal–conservative orientation (worldview beliefs)*	
Liberal	283 (68.9%)
Centre	80 (19.5%)
Conservative	48 (11.7%)

## Data Availability

Data generated as part of this study with replication codes for all analyses are available from the corresponding author upon reasonable request.

## Supplementary Materials

The following supporting information can be downloaded at: https://www.mdpi.com/article/10.3390/vaccines12040359/s1, Table S1. Students’ support for anti-vaccine theories by year of study.
