# Supplementary material for: Investigating Beliefs in Anti-Vax Conspiracy Theories among Medical Students
Source: Vaccines (Basel). 2024 Mar 27;12(4):359. doi: 10.3390/vaccines12040359 (PMC11054095; doi:10.3390/vaccines12040359)
Supplement: Supplementary file 1 [file vaccines-12-00359-s001.zip › vaccines-2897049-supplementary.pdf]

Supplementary material

Table S1. Students' support for anti-vaccine theories by year of study

Questions	Year of study	Definitely/rather not	Definitely/rather yes	Do not know
Vaccinations are an effective form of fighting infectious diseases	1	1 (0.9%)	105 (96.3%)	3 (2.8%)
	2	4 (3.1%)	127 (96.9%)	n.s.
	3	1 (1.2%)	80 (98.8%)	n.s.
	4	2 (2.8%)	69 (95.8%)	1 (1.4%)
	5	n.s.	27 (100%)	n.s.
Vaccines can cause autism	1	104 (95.4%)	1 (0.9%)	4 (3.7%)
	2	127 (96.9%)	1 (0.8%)	3 (2.3%)
	3	80 (98.8%)	n.s.	1 (1.2%)
	4	70 (97.2%)	n.s.	2 (2.8%)
	5	27 (100%)	n.s.	n.s.
HPV vaccines cause infertility	1	97 (89%)	n.s.	12 (11%)
	2	123 (93.9%)	1 (0.8%)	7 (5.3%)
	3	75 (92.6%)	n.s.	6 (7.4%)
	4	72 (100%)	n.s.	n.s.
	5	26 (96.3%)	1 (3.7%)	n.s.
mRNA vaccines can modify the human genome	1	97 (89.0%)	4 (3.7%)	8 (7.3%)
	2	121 (92.4%)	6 (4.6%)	4 (3.1%)
	3	78 (96.3%)	1 (1.2%)	2 (2.5%)
	4	71 (98.6%)	1 (1.4%)	n.s.
	5	25 (92.6%)	2 (7.4%)	n.s.
Vaccinations against COVID-19 serve depopulation	1	103 (94.5%)	2 (1.8%)	4 (3.7%)
	2	128 (97.7%)	1 (0.8%)	2 (1.5%)
	3	81 (100%)	n.s.	n.s.
	4	71 (98.6%)	n.s.	1 (1.4%)
	5	26 (96.3%)	1 (3.7%)	n.s.
COVID-19 vaccinations contain microchips	1	108 (99.1%)	n.s.	1 (0.9%)
	2	129 (98.5%)	1 (0.8%)	1 (0.8%)
	3	81 (100%)	n.s.	n.s.
	4	71 (98.6%)	n.s.	1 (1.4%)
	5	27 (100%)	n.s.	n.s.
The Zika, Ebola, MERS and SARS-CoV-2 epidemics were created to increase profits by biotechnology companies	1	94 (86.2%)	2 (1.8%)	13 (11.9%)
	2	120 (91.6%)	3 (2.3%)	8 (6.1%)
	3	76 (93.8%)	1 (1.2%)	4 (4.9%)
	4	69 (95.8%)	n.s.	3 (4.2%)
	5	26 (96.3%)	1 (3.7%)	n.s.

Note: [n.s.] response not selected
